# Total Hip Arthroplasty Following the Girdlestone Procedure in a Sickle Cell Disease Patient

**DOI:** 10.7759/cureus.65240

**Published:** 2024-07-24

**Authors:** Mohammad Alsaleem, Hassan A Al Abdrabalnabi, Bashayer F Al Furaikh, Nasser A Althafar

**Affiliations:** 1 Orthopedic Surgery, Al Moosa Specialist Hospital, Al-Ahsa, SAU; 2 Orthopedic Surgery, King Fahad Hospital in Al Hofuf, Al-Ahsa, SAU

**Keywords:** sickle cell disease, avascular necrosis, girdle-stone, orthopaedic, total hip replacement

## Abstract

Sickle cell disease often leads to avascular necrosis (AVN) of the hip joint, resulting in joint pain and restricted range of motion. In cases where traditional treatments like total hip arthroplasty or core decompression may not suffice, the Girdlestone procedure, involving the resection of the femoral head, is considered. This case study centers on a 19-year-old male nursing student with sickle cell disease who underwent a Girdlestone procedure at 16 years of age, seeking relief from hip pain and limited mobility. However, the procedure led to leg length discrepancy and reduced hip function. Subsequent total hip arthroplasty successfully converted the prior procedure into a stable joint, improving the patient's range of motion and eliminating pain. The comprehensive surgical approach, including soft tissue releases and postoperative rehabilitation, significantly enhanced the patient's quality of life, emphasizing the importance of total hip arthroplasty as a superior intervention post-Girdlestone procedure.

## Introduction

Sickle cell disease is one of the most common causes of avascular necrosis (AVN) of the hip joint [1]. To minimize joint pain, depending on the stage of avascular necrosis of the hip, physicians tend to go for invasive procedures such as total hip arthroplasty, core decompression, and in rare cases, Girdlestone procedure [2-3]. The girdlestone procedure involves resectioning the proximal part of the femur, specifically the head and neck [4]. The surgery involves the resection of the femoral head to the lesser trochanter, followed by approximation of the femur into the acetabulum, and finally the reattachment of associated soft tissue [5]. The primary goal of the Girdlestone procedure is pain relief [6]. Although the Girdlestone procedure provides adequate pain relief, it causes leg length discrepancy and, to some extent, restricted hip range of motion [7]. Furthermore, a 2-3 inch shortening is usually an expected outcome for which a total hip replacement is required following the procedure [8]. Total hip arthroplasty following the Girdlestone procedure is needed to improve the functionality of the hip joint [9].

## Case presentation

This 19-year-old male student in nursing school, known to have sickle cell disease, presented to our clinic complaining of right hip pain and restricted range of motion for four years, at the age of 16 years. Pain was related to certain hip movements and positions such as sitting on a low stool. The pain was aggravated by movement and relieved by analgesia and rest. The patient denied any history of steroid use, traumatic hip events, or alcoholism. The patient underwent the Girdlestone procedure four years ago at the age of 16 years in another hospital for the purpose of pain relief.

Upon local examination of the hip, there was no gluteal muscle wasting or contractures. The pelvis was tilted due to the compensation for the shortening of the right lower limb. Leg length discrepancy of 5 cm. The patient presented with axillary crutches with complete dependency on them. Furthermore, he couldn’t stand balanced on his feet due to shortening as his right foot did not reach the ground. He was not using a shoe lift orthotic as he had tried it before and found it uncomfortable. The patient had a short-limb gait. Palpation over the hip and groin showed no tenderness.

**Figure 1:**
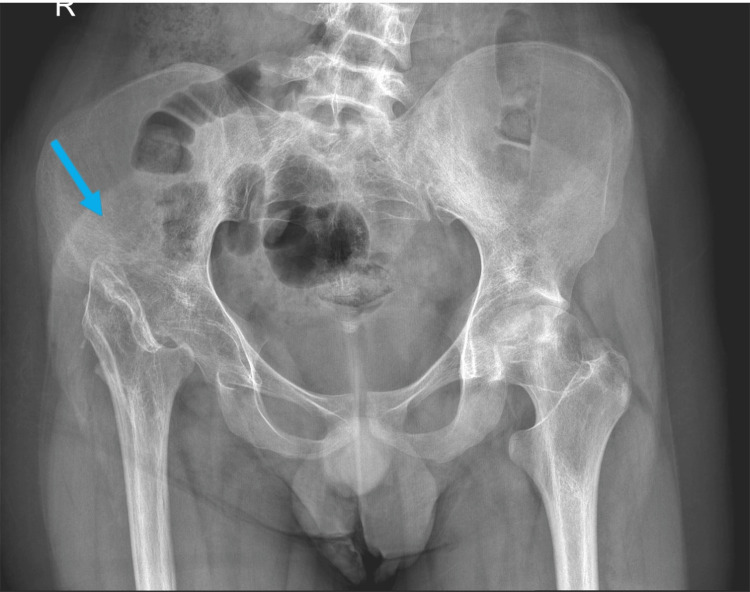
Pre-THR X-ray image of the pelvis THR: Total Hip Arthroplasty

The patient’s right hip range of motion was as follows; flexion (0-30), extension (0), abduction (0 to 20), adduction (0-20), external rotation (0-30), and internal rotation (0-5). For special tests, the Thomas test was done to identify muscle tightness and was positive. Furthermore, the Trendelberg test was positive, indicating a defective hip abductor mechanism. The patient had a Harris score of 50.9, which is interpreted as a poor outcome. Pulses of the femoral artery, popliteal artery, dorsalis pedis artery, and posterior tibialis were regular and intact. All sensory and motor functions of the femoral nerve, saphenous nerve and sciatic nerve were intact. On Investigation, X-ray showed right hip Girdlestone with high riding of the proximal femur. The morphology of the proximal femur was Durr A (Figure 1).

CT showed elongation of the greater trochanter following query iatrogenic fracture during the girdle stone procedure that was not addressed intra-operatively. Furthermore, the level of the osteotomy was just above the lesser trochanter. After recording the above-mentioned detailed history, examination, and investigations, we decided to perform a total hip arthroplasty to relieve the patient’s suffering and improve his function and quality of life. Risks and benefits were explained to the patient and his family.

**Figure 2:**
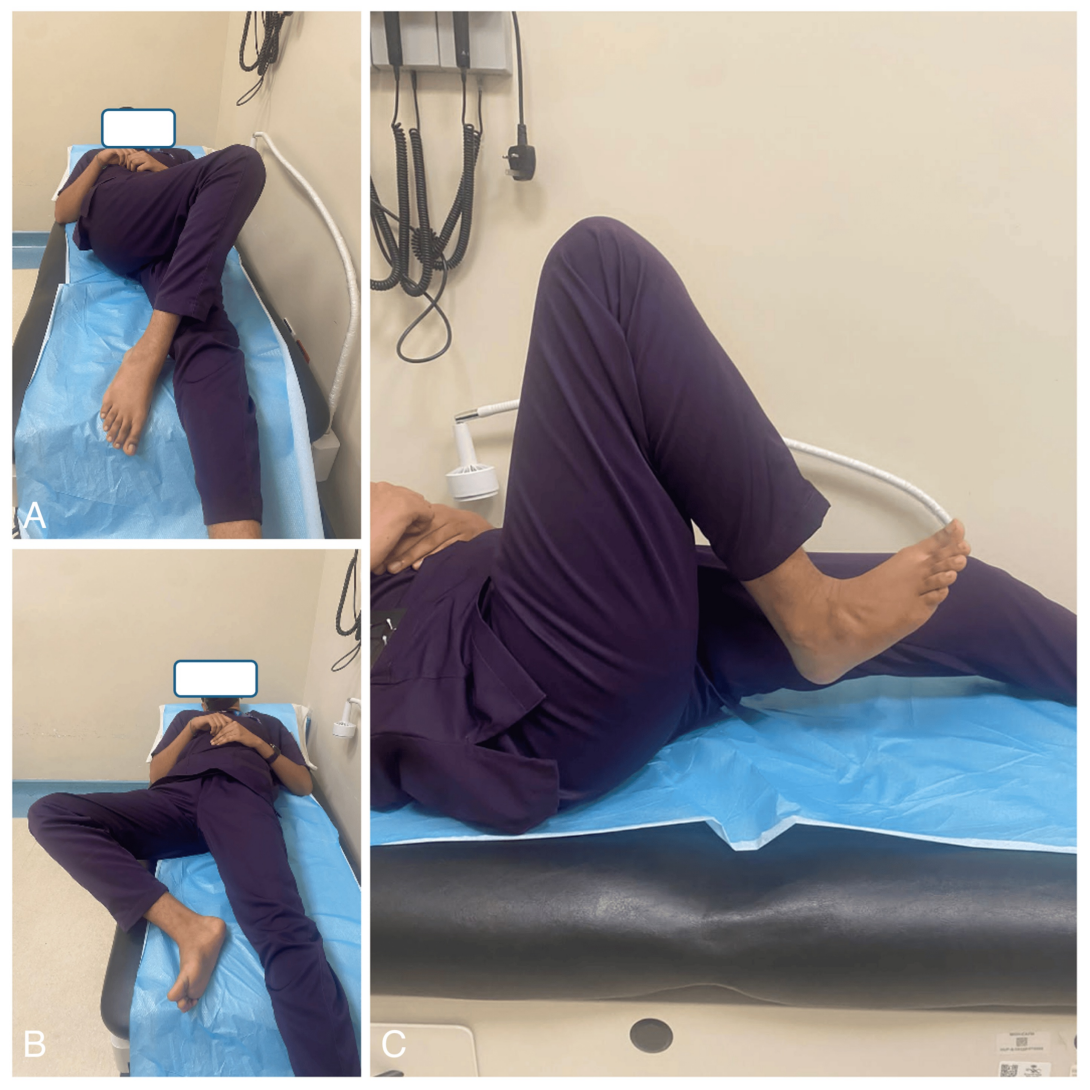
Post THR range of motion of the hip THR: Total Hip Arthroplasty. A: Right hip adduction; B: Right hip abduction; C: Right Hip flexion.

**Figure 3:**
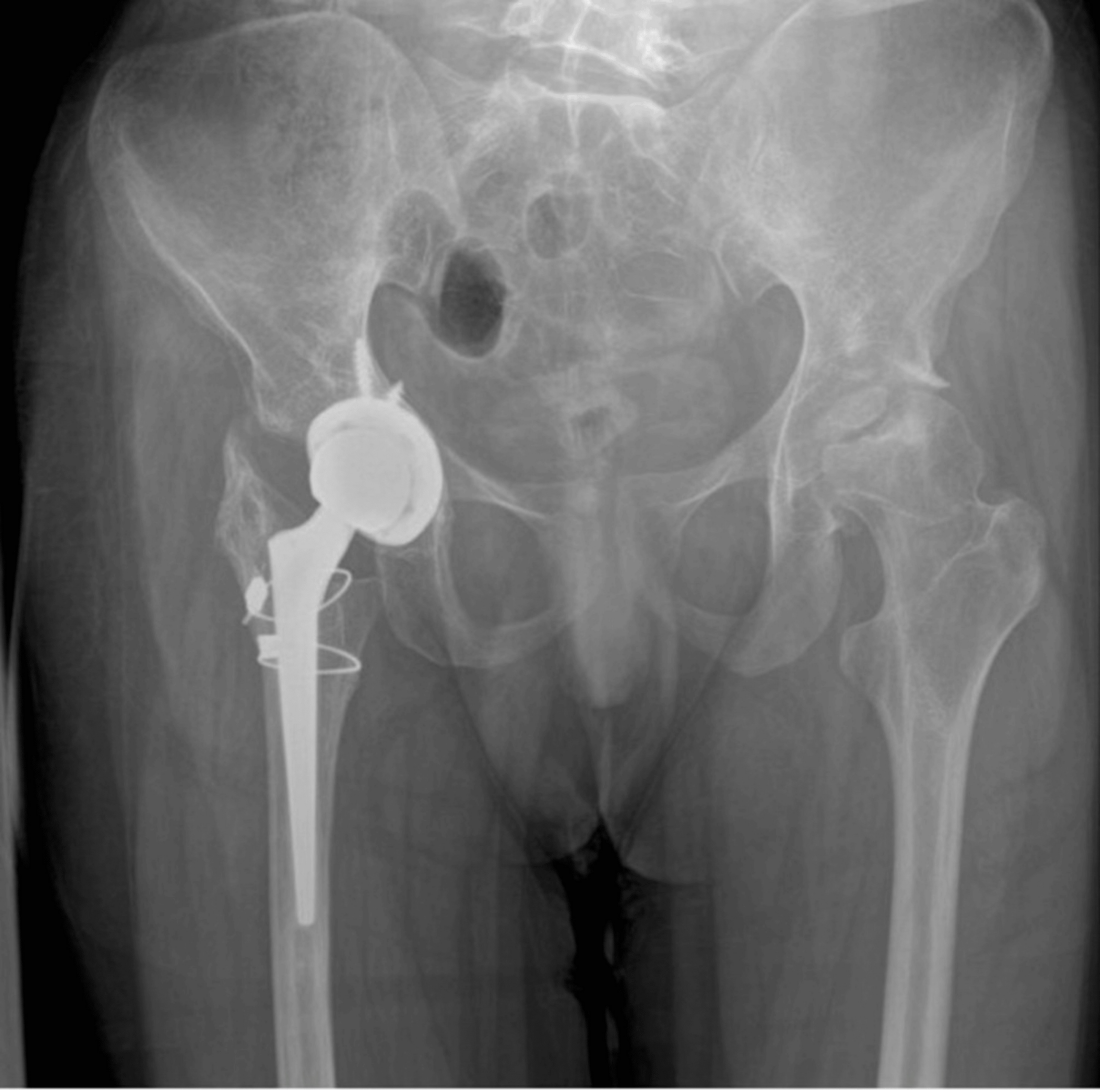
Immediate post-operative X-ray of the pelvis

The patient was admitted and optimized for surgery. In September of the year 2022, the patient was placed in the supine position where the adductor tenotomy was carried out due to tightness of the adductor muscle and limited abduction. Afterward, the patient was placed on the lateral position utilizing the modified Harding hip approach for meticulous soft tissue release and balancing. The soft tissue releases were as follows: gluteus maximus release, release of the entire anterior capsule, and iliopsoas tendon released from the lesser trochanter. Afterward, the true acetabulum was addressed through fluoroscopy. Reaming was done up to the size 48, a cup size 48 was inserted in the correct position and fixed with two acetabular screws. After completing the acetabular component, the proximal femur was addressed. The femoral canal was prepared. The canal was very tight (Durr A). Proximal reaming was done to open the canal. Sequential broaching was done till the stability of axial and rotation. A stress fracture had developed and two cerclage wires were applied. Reduction was attempted with difficulty and traction, and this was expected due to the high riding of the proximal femur. The hip was very stable in all directions. We found that there was no need for proximal osteotomy during surgery. The surgeon successfully converted the Girdlestone to a total hip arthroplasty (Figure 3). The procedure was uneventful, and the patient was later shifted to the ward in a stable state. On day 1 post-operatively, the patient was mobilized with crutches to toe touch the floor only with 25% of his weight. The patient was restricted to flexing the hip more than 90 degrees, external or internal rotation, and adducting the hip or crossing the legs. Physiotherapy was initiated in the first week to regain the hip’s range of motion and muscle power. Pain during the first two weeks post-operatively was controlled with analgesics. Extensive physiotherapy helped achieve gradual weight bearing on the limb. After the six-month interval, the patient was pain free, mobilizing well without any assistive aids. The hip’s range of motion has improved tremendously and the patient has become independent in his daily activities. The patient’s right hip range of motion today is as shown in Figure 2; flexion (0-90), extension (0), abduction (0- 80), adduction (0-30), external rotation (0-60), and internal rotation (0-80). Muscle power was 5/5 and is comparable to the contralateral limb. The Harris score post-operatively was 92.8 which is an excellent outcome of the procedure (Figure 3).

## Discussion

Avascular necrosis of the hip joint among sickle cell disease patients has been a major issue in the region [10]. Tackling symptomatic avascular necrosis of the hip can be done by the following procedures: core decompression, rotational osteotomy, vascularized free fibular transfer, total hip replacement, total hip resurfacing, and hip arthrodesis [11]. Girdlestone procedure was described by Gathrone Robert Girdlestone in 1928 [12]. Gathorne introduced the Girdlestone procedure as a salvage procedure for the pyogenic infected hips to relieve pain and irradicate infection [13]. Although pain relief is achievable in some cases following the Girdlestone procedure, in most cases, the result is either temporary or not satisfactory [14]. Interestingly, sickle cell disease patients show no promising results regarding pain relief after the Girdlestone procedure [15]. The procedure is rarely done on younger populations suffering from avascular necrosis because it lacks functional outcomes in comparison to total hip arthroplasty [14]. Furthermore, younger patients have higher functional demands and expectations, so they are likely to be dissatisfied with the operation [15]. Our patient suffered with his nearly immobile hip following the Girdlestone procedure and was seeking a solution. Upon his visits, history given and physical examination, a total hip arthroplasty decision was made. Although it is considered a difficult procedure with multiple risks, the patient’s functional well-being was a priority to continue his education and enjoy life’s adventures. We reviewed the literature on the results of the Girdlestone procedure followed by total hip arthroplasty in young Sickler patients. There are no reports of total hip replacement as in our case report.

In our patient, a total hip arthroplasty following the Girdlestone procedure resulted in an excellent functional outcome and patient satisfaction after two years of follow-up. Although the Girdlestone procedure might have been indicated priorly to improve the patient’s pain, it caused significant activity limitation, leg length discrepancy, and recurrence of pain secondary to femur migration as illustrated in Figure 1.

Research indicates that individuals who have undergone the Girdlestone procedure exhibit a greater pre-total hip arthroplasty leg length discrepancy compared to those undergoing revision total hip replacement (THR) [16]. The optimal outcome in terms of elongation, around 3 cm, was found to minimize the risk of sciatic nerve injury during the procedure [16]. The lateral approach prevents damage to the external rotator muscles which maintains muscle strength to avoid postoperative dislocation [17]. Furthermore, the adductor tenotomy, soft tissue releases, and balancing were crucial to retain the functionality of the joint and leg length and restoring normal gait [17]. Additionally, the postoperative rehabilitation extensive program aids to improve the function of the implanted joint. Furthermore, physiotherapy sessions were focused on muscle strengthening, gradual weight bearing, increasing the range of motion and stretching exercises [18]. The most common complications following a total hip replacement are dislocations, infections, neurovascular injuries, thromboembolic disease, and peri-prosthetic fractures [19]. Moreover, the rate of these complications increases in the 90-day period post-operatively [20]. In this current case, the patient was following a detailed program that consisted of multiple steps. First, the patient was counseled about the risk of persistent limp, dislocation, and occurrence of infection after conversion. Second, meticulous and careful dissection and intra-operative soft tissue handling are necessary to avoid neurovascular compromise. As well as gentle and proper placement of surgical instruments deep in the joint. The previously mentioned soft tissue releases and the acetabulum's anatomical reconstruction promoted the limb's lengthening and restored the abductor muscles' balance and strength [17]. Furthermore, gentle dislocation and relocation were done by the most senior in the team to avoid peri-prosthetic fractures. Third, anti-coagulation daily for 21 days, followed by anti-platelet for another 21 days to avoid thromboembolic events. Fourth, restriction of external rotation, internal rotation, flexion more than 90 degrees, and adduction of the hip to avoid dislocation in the 90-day period. Lastly, maintenance of the implant integrity was achieved through gradual weight bearing. The above-mentioned holistic approach to such patients showed excellent results and increased the patient’s Harris score from 50.9 to 92.8 [21]. Moreover, it has been shown that satisfaction after total hip arthroplasty increases with gait improvement, pain relief and functional improvement [22,23].

## Conclusions

In conclusion, the Girdlestone procedure is a salvage procedure that is usually a last resort in case of infected joints or prostheses due to its result being either temporary or not satisfactory, especially among the young active population. A total hip arthroplasty following a Girdlestone procedure is the best choice to restore the functionality of the joint and improve quality of life. A detailed meticulous surgical approach followed by proper cautious physiotherapy and anti-coagulation shows promising results.
